# The role of circRNAs in regulation of drug resistance in ovarian cancer

**DOI:** 10.3389/fgene.2023.1320185

**Published:** 2023-12-13

**Authors:** Jun Zhan, Zhiyi Li, Changsheng Lin, Dingding Wang, Lei Yu, Xue Xiao

**Affiliations:** ^1^ Department of Obstetrics and Gynecology, West China Second University Hospital, Sichuan University, Chengdu, Sichuan, China; ^2^ Key Laboratory of Birth Defects and Related Diseases of Women and Children, Ministry of Education, Sichuan University, Chengdu, Sichuan, China

**Keywords:** chemotherapy, circRNA, drug, miRNA, ovarian cancer, resistance

## Abstract

Ovarian cancer is one of the female reproductive system tumors. Chemotherapy is used for advanced ovarian cancer patients; however, drug resistance is a pivotal cause of chemotherapeutic failure. Hence, it is critical to explore the molecular mechanisms of drug resistance of ovarian cancer cells and to ameliorate chemoresistance. Noncoding RNAs (ncRNAs) have been identified to critically participate in drug sensitivity in a variety of human cancers, including ovarian cancer. Among ncRNAs, circRNAs sponge miRNAs and prevent miRNAs from regulation of their target mRNAs. CircRNAs can interact with DNA or proteins to modulate gene expression. In this review, we briefly describe the biological functions of circRNAs in the development and progression of ovarian cancer. Moreover, we discuss the underneath regulatory molecular mechanisms of circRNAs on governing drug resistance in ovarian cancer. Furthermore, we mention the novel strategies to overcome drug resistance via targeting circRNAs in ovarian cancer. Due to that circRNAs play a key role in modulation of drug resistance in ovarian cancer, targeting circRNAs could be a novel approach for attenuation of chemoresistance in ovarian cancer.

## Introduction

Ovarian cancer is one of the malignant tumors, which belongs to the female reproductive system tumors (Giaquinto et al., 2022; Siegel et al., 2023). Ovarian cancer goes undetected in its early stages because its symptoms are vague and similar to other common female reproductive diseases. The symptoms include bloating, pelvic or abdominal pain, and changes in urinary habits. Therefore, ovarian cancer has the high fatality rate (Torre et al., 2018). The most common ovarian cancer is epithelial ovarian cancer, and the other types include stromal tumors and germ cell tumors (Arnaoutoglou et al., 2023). It is unknown regarding the cause of ovarian cancer development; however, several risk factors could contribute to ovarian carcinogenesis, such as a family history of ovarian cancer, age and certain reproductive factors (Ali et al., 2023; Rousseau et al., 2023). The reproductive factors include early onset of menstruation, late menopause and never having been pregnant. The treatment options for ovarian cancer have surgery, radiation therapy and chemotherapy (Konstantinopoulos and Matulonis, 2023; Matsas et al., 2023). Because drug resistance is one important cause of therapeutic failure, it is pivotal to explore the drug resistant mechanisms and to overcome drug resistance in ovarian cancer (Pawlowska et al., 2023).

Genetic mutations have been reported to associate with ovarian cancer progression, such as BRCA1 (breast cancer 1) mutation and BRCA2 (breast cancer 2) mutation (Sanchez-Lorenzo et al., 2022). Moreover, epigenetic regulation has been revealed to participate in the development of ovarian cancer (Wang et al., 2022a; Zhao et al., 2022). It is known that epigenetics refers to heritable changes and do not change DNA sequence. Instead, epigenetic changes include DNA methylation, chromatin remodeling and histone protein modification, which affect gene activity (Portela and Esteller, 2010). Epigenetic alterations often cause abnormal activation or inactivation of key genes, leading to various diseases (Carter and Zhao, 2021; Van Loo et al., 2022). Hypermethylation of promoter regions contributes to the silencing of tumor suppressor genes, while hypomethylation of certain regions in oncogenes confers the activation of oncogenes (Yousefi et al., 2022; Greenberg and Bourc’his, 2019). Chemical modifications of histone proteins affect the DNA accessibility to the transcriptional complex, including phosphorylation, ubiquitination, methylation and acetylation. Abnormal histone modifications lead to the dysregulation of gene expression and cause various diseases. Chromatin remodeling can reposition nucleosomes and regulate the DNA accessibility for transcription (Millan-Zambrano et al., 2022; Shvedunova and Akhtar, 2022).

NcRNAs have been documented to regulate carcinogenesis in a variety of human cancers (Winkle et al., 2021; Liu and Shang, 2022; Zhang et al., 2023a). It is clear that ncRNAs do not code for proteins, but regulate gene expression and cellular functions. NcRNAs include miRNAs (microRNAs), lncRNAs (long noncoding RNAs), snoRNAs (small nucleolar RNAs), piRNAs (piwi-interacting RNAs), rRNAs (ribosomal RNAs), siRNAs (small interfering RNAs) and circRNAs (circular RNAs) (Anastasiadou et al., 2018). In general, miRNAs have around 22 nucleotides in length and bind to the mRNAs of target genes, contributing to their degradation or inhibition of translation (Treiber et al., 2019; Shang et al., 2023). LncRNAs have more than 200 nucleotides and perform diverse functions, such as modulating chromatin structure, regulating gene expression, acting as scaffolds for protein complexes (Liu et al., 2021a; Mattick et al., 2023). CircRNAs are covalently closed RNA molecules, which are highly stable. CircRNAs can sponge miRNAs and sequester miRNAs, which prevent miRNAs from regulation of their target mRNAs. In addition, circRNAs can interact with DNA or proteins to modulate gene expression (Chen, 2020; Kristensen et al., 2022). It has been documented that ncRNAs regulate drug sensitivity in ovarian cancer (Zhang et al., 2021a; Liu et al., 2022a). Understanding the complex interplay between noncoding RNAs and drug resistance in ovarian cancer is critical for developing more effective diagnostic tools and therapeutic strategies.

## Drug resistance

Drug resistance have obtained much attention to contribute to cancer treatment failure (Freedy and Liau, 2021). Drug resistance refers to the ability of tumor cells to survive in the presence of therapies, such as targeted therapy, chemotherapy and immunotherapy (Pisa and Kapoor, 2020). Drug resistance occurs through various molecular mechanisms and limits the effectiveness of cancer therapies. Inherent resistance might be because of genetic mutations or alterations, which cause the tumor cells less susceptible to drugs. Acquired resistance develops over time after tumor cells adapt to the therapy, leading to therapy failure (Ye et al., 2023). The potential mechanisms of drug resistance include genetic mutations, activation of alternative pathways, efflux pumps, enhanced DNA repair capabilities, tumor microenvironment change (Liu et al., 2022b; Bou Antoun and Chioni, 2023). Epigenetic changes, including ncRNAs, have been reported to affect gene expression and contribute to drug resistance (Jiang et al., 2020; Xie et al., 2022).

Chemotherapy often elicits a good initial benefit; however, some patients with ovarian cancer develop malignant progression due to that tumor cells obtain resistance to chemotherapeutic drugs. Evidence has suggested that circRNAs could play an oncogenic role or exert tumor suppressive functions in ovarian cancer (Zhang et al., 2022a; Ghafouri-Fard et al., 2022; Najafi, 2023). Moreover, circRNAs might be biomarkers for diagnosis and prognosis in ovarian cancer (Qiu et al., 2022). For instance, circRNA1656 was reported to act as a potential biomarker in high grade serous ovarian cancer (Gao et al., 2019a). Plasma circN4BP2L2 was found as a useful biomarker for diagnosis in epithelial ovarian cancer (Ning et al., 2022). Furthermore, circRNAs have been identified to regulate drug resistance in ovarian cancer (Lan et al., 2021). In addition, drug resistance changes the expression of certain circRNAs (Qin et al., 2023a). However, the role and molecular mechanism of circRNAs in ovarian cancer are elusive (Liu et al., 2022c). In this review, we will briefly discuss the role of circRNAs in the development of ovarian cancer. Moreover, we will discuss the function and mechanisms of circRNAs on drug resistance in ovarian cancer. Furthermore, we will describe the strategies to overcome drug resistance via targeting circRNAs in ovarian cancer.

## CircRNAs in ovarian cancer progression

Mounting evidence has revealed that circRNAs critically regulate ovarian cancer development and progression (Yang et al., 2020; Foruzandeh et al., 2021). The circ-ITCH inhibited tumor progression via regulation of miR-145/RASA1 and miR-106a/CDH1 in ovarian cancer (Hu et al., 2018; Lin et al., 2020). CircRNA UBAP2 sponged miR-144, elevated the expression of CHD2, and facilitated tumor progression in ovarian cancer (Sheng et al., 2019). Xu et al. reported that circ-UBAP2 controlled the miR-382-5p/PRPF8 axis and enhanced cell proliferation and inhibited cell apoptosis in ovarian cancer (Xu et al., 2020a). CircPLEKHM3 regulated the expression of miR-9, BRCA1, KLF4, Akt1, DNAJB6 and performed a tumor suppressive function in ovarian cancer (Zhang et al., 2019a). CircWHSC1 sponged miR-145 and miR-1182 and upregulated the expression of MUC1 and hTERT, leading to promotion of tumor progression in ovarian cancer (Zong et al., 2019). CircPUM1 enhanced oncogenesis and progression via interaction with miR-615-5p and miR-6753-5p in ovarian cancer (Guan et al., 2019). Circ_100395 repressed tumor metastasis and proliferation via targeting miR-1228/p53/EMT (epithelial-mesenchymal transition) axis in ovarian cancer (Li et al., 2020a). CircMUC16 interacted with miR-199a and ATG13, and accelerated autophagy in epithelial ovarian cancer (Gan et al., 2020). Circ0004390 sponged miR-198 and elevated the expression of MET and facilitated cell proliferation in ovarian cancer (Xu et al., 2020b). Circ_102958 facilitated tumor progression via modulation of miR-1205 and SH2D3A expression in ovarian cancer (Wang et al., 2020a).

CircRNA FGFR3 triggered EMT by sponging miR-29a-3p and upregulating E2F1 expression in ovarian cancer (Zhou et al., 2020a). CircRNA-9119 regulated the miR-21-5p/PTEN/Akt pathway and attenuated cell viability in ovarian cancer (Gong et al., 2020). Circ_0072995 accelerated the tumor progression via regulating miR-147a and CDK6 expression in epithelial ovarian cancer (Ding et al., 2020). In addition, circKRT7/miR-29a-3p/COL1A1 axis was critically involved in promotion of ovarian cancer cell progression (An et al., 2020). Wang et al. found that circ-0001068 could be a biomarker for ovarian cancer and induce the expression of PD1 expression in T cells (Wang et al., 2020b). CircRNA PVT1 controlled cell invasion and proliferation through targeting FOXM1 expression by sponging miR-149-5p in ovarian cancer (Li et al., 2021). CircRAB11FIP1 induced autophagy flux via regulation of miR-129 and DSC1 in ovarian cancer (Zhang et al., 2021b). Circ-PTK2 (hsa_circ_0008305) targeted miR-639 and FOXC1 expressions and governed the pathogenic processes in ovarian cancer (Wu et al., 2021a).

Circ_0000554 was reported to increase cell invasion and proliferation via sponging miR-567 in ovarian cancer (Wang et al., 2022b). Both circATRNL1 and circZNF608 were found to suppress ovarian cancer progression via sequestering miR-152-5p and regulating encoding protein (Lyu et al., 2022). CircCERS6 was revealed to inhibit tumor development via modulation of miR-630 and RASSF8 in epithelial ovarian cancer (Li et al., 2022a). Wu et al. discovered that circFBXO7/miR-96-5p/MTSS1 axis played a pivotal role in regulation of the Wnt signaling pathway in ovarian cancer (Wu et al., 2022). Circ-BNC2 (hsa_circ_0008732) was uncovered to regulate the miR-223-3p and FBXW7 expressions and retard tumor progression in ovarian cancer (Liu et al., 2022d). Hsa_circ_0001445 was reported to act as a tumor suppressor via regulation of miR-576-5p/SFRP1 axis in ovarian cancer (Wu et al., 2023a). Circ_0015756 can influence the miR-145-5p/PSAT1 axis and lead to enhancement of ovarian cancer progression (Pan et al., 2022a). Extracellular vesicle-packaged circATP2B4 enhanced tumor metastasis via induction of M2 macrophage polarization through interaction with miR-532-3p and regulation of SREBF1 expression in epithelial ovarian cancer (Wang et al., 2023a). Similarly, exosome-transmitted circIFNGR2 influenced tumor metastasis via affecting the miR-378/ST5 axis in ovarian cancer (Chen et al., 2023a). Downregulation of circMFN2 reduced tumor progression and regulated glycolysis via affecting miR-198 and CUL4B pathway in ovarian cancer (Song et al., 2023). Hsa_circ_0001741 adsorbed the miR-188-5p and increased the expression of FOXN2, contributing to inhibition of cell proliferation in ovarian cancer (Wang et al., 2023b). Lastly, circ-PHC3 overexpression stimulated ovarian cancer progression by sponging the miR-497-5p and upregulating SOX9 pathway (Wang et al., 2023c). Altogether, circRNAs participate in ovarian cancer development and progression.

## Role of circRNAs in drug resistance

### CircRNAs promote paclitaxel resistance

#### CircCELSR1

One study explored circRNA expression profiles in 54 ovarian cancer tissues and 54 normal controls, and identified them using circRNA-sequencing based profiles in 4 ovarian tumor specimens and 4 normal ovarian controls. 4388 circRNAs (2556 upregulated, 1832 downregulated) were differentially expressed in the ovarian cancer tissues vs. normal controls. The expression of six circRNAs, including circCELSR1, circEXOC6B, circBNC2, circRHOBTB3, circFAM13B and circN4BP2L2, was associated with clinicopathological characterizations of ovarian cancer. CircEXOC6B and circN4BP2L2 expression could be diagnostic and prognostic biomarkers for ovarian cancer patients (Ning et al., 2018). However, this study did not explore the association between circEXOC6B, circN4BP2L2 and drug resistance in ovarian cancer (Ning et al., 2018). Another study showed that knockdown of circCELSR1 reduced invasion, migration, proliferation and EMT and stimulated apoptosis via binding to miR-598 and upregulation of BRD4 in ovarian cancer cells. Inhibition of miR-598 or overexpression of BRD4 abrogated the function of circCELSR1 in ovarian cancer. Silencing of circCELSR1 decreased tumor metastasis and reduced tumor growth in nude mice (Zeng et al., 2020).

Zhang et al. reported that circCELSR1 (hsa_circ_0063809) conferred paclitaxel (PTX) resistance by influencing the expression of FOXR2 via targeting miR-1252 (Zhang et al., 2020a). Microarray analyses were performed to detect the expression of circCELSR1 in five PTX-sensitive ovarian tissues and five PTX-resistant ovarian cancer tissues. It was found that circCELSR1 was highly expressed in PTX-resistant ovarian cancer tissues. Moreover, qRT-PCR was performed to measure the expression of circCELSR1 in 36 ovarian cancer tissues and adjacent non-tumor tissues (Zhang et al., 2020a). Higher expression of circCELSR1 was displayed in ovarian cancer species. Cell counting kit-8 (CCK-8) data showed that silencing of circCELSR1 promoted PTX-mediated cytotoxicity in ovarian cancer cells. Moreover, suppression of circCELSR1 induced cell apoptosis and led to cell cycle arrest at G0/G1 phase in ovarian cancer cells (Zhang et al., 2020a). *In vivo* results revealed that inhibition of circCELSR1 suppressed tumor growth of ovarian cancer. Mechanistically, circCELSR1 was revealed to sponge miR-1252 and subsequently inhibit the expression of FOXR2 in ovarian cancer cells. Hence, circCELSR1 promoted PTX resistance via targeting miR-1252/FOXR2 axis in ovarian cancer (Zhang et al., 2020a).

Similarly, Wei et al. found that circCELSR1 expression was increased in PTX-resistant ovarian cancer cells and tumor specimens. Knockdown of circCELSR1 caused inhibition of cell viability, colony formation and promoted cell apoptosis and PTX sensitivity and reduced cell cycle progression in PTX-resistant ovarian cancer cells. Moreover, circCELSR1 sponged miR-149-5p and upregulated SIK2 (salt inducible kinase 2) expression. Suppression of miR-149-5p rescued the effects of circCELSR1 silencing on PTX resistance in PTX-resistant ovarian tumor cells. Meanwhile, upregulation of miR-149-5p blocked PTX resistance via targeting SIK2 in PTX-resistant ovarian cancer cells. In summary, circCELSR1 could target miR-149-5p/SIK2 axis and facilitated PTX resistance in ovarian cancer (Wei et al., 2021a).

#### CircANKRD17

ZEB1 (Zinc finger E-box-binding homeobox 1) has been known to trigger the EMT process in human cancer types (Wang et al., 2022c; Park et al., 2022; Perez-Oquendo and Gibbons, 2022). The transcription factor ZEB1 has been reported to be involved in EMT in ovarian cancer (Wei et al., 2021b). Zhang et al. found that circular RNA S-7 mediated EMT progression via sponging miR-641 and elevating ZEB1 and MDM2 in ovarian cancer (Zhang et al., 2020b). Cui et al. reported that ZEB1 promoted cisplatin resistance via inhibition of SLC3A2 expression in ovarian cancer (Cui et al., 2018). LncRNA NEAT1 conferred paclitaxel resistance via regulation of miR-194/ZEB1 axis in ovarian cancer (An et al., 2017). Similarly, inhibition of miR-429 led to drug resistance development via influencing ZEB1 expression in epithelial ovarian cancer (Zou et al., 2017). These findings suggested that ZEB1 was involved in drug resistance of ovarian cancer. One group showed that ZEB1 promoted the transcription of circANKRD17 and caused the progression of ovarian cancer (Cai and Zhang, 2022). Inhibition of ZEB1 reduced cell migration, invasion, EMT and proliferation in ovarian cancer. Silencing of ZEB1 downregulated the expression of circANKRD17, while overexpression of ZEB1 upregulated circANKRD17 expression level in ovarian cancer cells. ZEB1-mediated tumor promotion was due to upregulation of circANKRD17 in ovarian cancer (Cai and Zhang, 2022). Another group reported that circANKRD17 (circ_0007883) contributed to paclitaxel resistance via binding with FUS (fused in sarcoma) to make FOXR2 stability (Liang et al., 2023). The higher expression of circANKRD17 was seen in ovarian cancer tissues and ovarian tumor cell lines, which was associated with PTX sensitivity. In ovarian cancer cells, silencing of circANKRD17 induced apoptosis and decreased cell viability and increased PTX sensitivity. Moreover, circANKRD17 can bind with FUS, one RNA-binding protein, to stabilize FOXR2 (Liang et al., 2023). Therefore, circANKRD17 facilitated PTX resistance in ovarian cancer cells via interaction with FUS and maintenance of FOXR2 stability.

#### Circ_0061140

Several studies have revealed that circ_0061140 participated in the development and progression of various types of human cancers. For example, in endometrial carcinoma cells, circ_0061140 promoted tumor progression through impacting miR-149-5p/STAT3 (Liu et al., 2020a). In prostate cancer, circ_0061140 targeted miR-1193 and facilitated tumor malignant development (Wang et al., 2021a). In lung adenocarcinoma, circ_0061140 regulated miR-653 expression and elevated hexokinase 2 levels, leading to promotion of hypoxia-mediated migration, invasion and glycolysis (Wang et al., 2022d). In ovarian cancer, silencing of circ_0061140 abolished FOXM1-induced metastasis and cell proliferation via sponging miR-370 (Chen et al., 2018). One study showed that circ_0061140 conferred tumor malignant development via sponging miR-361-5p and upregulating the RAB1A expression in ovarian cancer cells (Zhang et al., 2022b). Another study reported that circ_0061140 affected miR-761/LETM1 (leucine zipper and EF-hand containing transmembrane protein 1) signaling and led to tumor progression in ovarian cancer (Ma et al., 2023). Moreover, circ_0061140 was found to be highly expressed in PTX-resistant ovarian cancer samples. Inhibition of circ_0061140 improved PTX sensitivity and induced apoptosis as well as suppressed invasion, proliferation and migration in ovarian cancer cells (Zhu et al., 2021). Downregulation of miR-136 restrained circ_0061140 knockdown-mediated PTX sensitivity and antitumor activity in ovarian cancer cells. Strikingly, downregulation of circ_0061140 retarded tumor growth and increased sensitivity to PTX treatment in mice. Furthermore, chromobox 2 (CBX2) was found to be a target of miR-136 in ovarian cancer. To summarize, circ_0061140 sponged miR-136 and elevated the expression of CBX2 in ovarian cancer, leading to PTX resistance and tumor malignant progression (Zhu et al., 2021).

#### Circ_0025033

Studies have shown the functions of circ_0025033 on colony formation, migration, invasion and glycolysis metabolism in ovarian cancer cells (Hou and Zhang, 2021). Ovarian cancer tissues and tumor cells have a higher expression of circ_0025033. Depletion of circ_0025033 reduced cell invasion, migration, glycolysis metabolism and colony formation in ovarian cancer cells. Mechanically, circ_0025033 influenced the expression of LSM4 via sponging miR-184 in ovarian cancer cells. Overexpression of LSM4 abolished the circ_0025033 knockdown-mediated inhibitory functions on cell invasion, colony formation, migration and glycolysis metabolism in ovarian cancer (Hou and Zhang, 2021). Another study showed that circ_0025033 was a key modulator in ovarian carcinogenesis. The expression of circ_0025033 was elevated in ovarian cancer tissues. Depletion of circ_0025033 led to suppression of proliferation, angiogenesis, and glutamine metabolism in ovarian cancer cells, and reduced tumor growth in mice. Notably, circ_0025033 promoted ovarian oncogenesis via sponging miR-370-3p and subsequent upregulation of SLC1A5. In parallel, miR-370-3p targeted the expression of SLC1A5 in ovarian cancer cells (Ma et al., 2022).

The involvement of circ_0025033 in regulating PTX resistance via targeting miR-532-3p and FOXM1 has been reported in ovarian cancer. In ovarian cancer tissues and tumor cells, there were high expression of circ_0025033 and FOXM1 and lower expression of miR-532-3p (Huang et al., 2022). After knockdown of circ_0025033 in PTX-resistant ovarian cancer cells, cell invasion and migration were inhibited, and apoptosis was induced. 3-(4, 5-Dimethylthiazol-2-yl)-2, 5-diphenyltetrazolium bromide (MTT) assay data showed that circ_0025033 downregulation induced PTX sensitivity in PTX-resistant ovarian cancer cells. Regarding mechanism, miR-532-3p and FOXM1 were involved in circ_0025033-induced PTX resistance in ovarian cancer (Huang et al., 2022).

#### CircATL2

CircATL2 (circRNA Atlastin GTPase 2) was found to be highly expressed in PTX-resistant ovarian cancer tissues and ovarian tumor cells. Silencing of circATL2 led to suppression of colony formation and induced cell apoptosis and cell cycle arrest in PTX-resistant ovarian cancer cells (Ying et al., 2022). Deletion of circATL2 attenuated the IC50 of PTX in ovarian cancer cells with PTX resistance. Moreover, *in vivo* data showed that knockdown of circATL2 restrained cell resistance to PTX in mice. Mechanistic study showed that circATL2 sponged miR-506-3p in PTX-resistant ovarian cancer cells (Ying et al., 2022). Consistently, knockdown of miR-506-3p abrogated the impacts of circATL2 silencing-mediated PTX sensitivity. Furthermore, NFIB acted as a downstream target of miR-506-3p. In addition, upregulation of miR-506-3p reduced cell resistance to PTX in ovarian cancer cells, which can be reversed by elevation of NFIB. In summary, circATL2 facilitated PTX resistance via affecting miR-506-3p/NFIB axis (Ying et al., 2022).

#### Circ_0000714

Guo et al. reported that downregulation of circ_0000714 governed RAB17 expression via sponging miR-370-3p and reduced PTX resistance in A2780 ovarian cancer cells. Moreover, RAB17 expression was upregulated in PTX-resistant A2780 ovarian cancer cells (Guo et al., 2020). Silencing of RAB17 enhanced PTX sensitivity, induced cell cycle arrest at G1 phase and attenuated cell proliferation in PTX-resistant A2780 cells. RAB17 activated the CDK6 and RB pathways and performed its biological behaviors. In addition, miR-370-3p was found to target RAB17 expression. Notably, circ_0000714 worked as a miR-370-3p sponge to regulate the expression of RAB17 in ovarian cancer cells. In conclusion, circ_0000714 targeted miR-370-3p/RAB17 axis and activated CDK6/RB signaling pathway, leading to reduction of paclitaxel resistance in ovarian cancer (Guo et al., 2020).

#### CircSETDB1

CircRNA SET domain bifurcated histone lysine methyltransferase 1 (circSETDB1) has been reported to regulate ovarian tumorigenesis. The expression level of circSETDB1 was increased in serous ovarian cancer tissues compared with normal fallopian tube tissues. CircSETDB1 expression was elevated in ovarian cancer cells in comparison with normal ovarian epithelial cells (Li and Zhang, 2021). Inhibition of circSETDB1 increased cell apoptosis and reduced cell migration, invasion and growth in serous ovarian cancer. Knockdown of circSETDB1 impaired tumor growth in mice. CircSETDB1 knockdown-induced malignant progression was blocked by inhibition of miR-129-3p. MAP3K3 (mitogen-activated protein kinase kinase kinase 3) was a target of miR-129-3p in serous ovarian cancer cells (Li and Zhang, 2021). Similarly, another group identified the higher expression of circSETDB1 in PTX-resistant ovarian cancer (Huang et al., 2023). Silencing of circSETDB1 exhibited anticancer activity via inhibition of proliferation and cell cycle process and induction of cell apoptosis in PTX-resistant ovarian cancer cells. Moreover, circSETDB1 targeted miR-508-3p and increased the expression of ABCC1 (ATP-binding cassette subfamily C member 1) in ovarian cancer cells. Blockade of miR-508-3p or upregulation of ABCC1 impaired the effects of circSETDB1 depletion on cell resistance to PTX in ovarian cancer. Mouse study also validated that circSETDB1 downregulation promoted cell sensitivity to PTX. In conclusion, circSETDB1 enhanced PTX resistance via inhibition of miR-508-3p and upregulation of ABCC1 expression (Huang et al., 2023).

Wang et al. discovered that serum circSETDB1 could be a potential biomarker for prediction of chemotherapy response and relapse in ovarian cancer. This group measured serum circSETDB1 levels in 18 chemo-resistant patients (platinum-taxane-combined chemotherapy), 42 chemo-sensitive patients and 60 healthy volunteers. In comparison with healthy controls, serum circSETDB1 expression was elevated in serous ovarian cancer patients (Wang t al., 2019a). CircSETDB1 expression level can distinguish ovarian cancer patients from healthy people. Lymph node metastasis, advanced FIGO (international federation of gynecology and obstetrics) stage and survival were correlated with high expression of circSETDB1 in ovarian cancer patients. Ovarian cancer patients with chemoresistance had a higher expression levels of serum circSETDB1, which can distinguish ovarian cancer patients with chemoresistance from those with chemosensitivity (Wang et al., 2019a).

#### Circ_0000231

Hsa_circ_0000231 has been revealed to participate in the progression of colorectal cancer cells. The expression of circ_0000231 was elevated in colorectal cancer specimens. Depletion of circ_0000231 alleviated the glycolysis, suppressed invasion, migration and growth via sponging miR-502-5p and upregulating MYO6 in colorectal cancer cells (Liu et al., 2020b). Moreover, a study indicated that sevoflurane, a volatile anesthetic, reduced the process of colorectal cancer via inhibition of circ_0000231 expression and upregulation of miR-622 expression in colorectal cancer cells. Colorectal cancer tissues had an upregulation of circ_0000231 and a downregulation of miR-622. In addition, circ_0000231 can sponge miR-622 and reduce its expression level. Sevoflurane inhibited cell migration, invasion and proliferation and induced apoptosis in colorectal cancer (Wang et al., 2021b). Liu et al. reported that circ_0000231 induced paclitaxel resistance via regulation of miR-140 and RAP1B in ovarian cancer. PTX-resistant ovarian cancer tissues and cells had an upregulation of circ_0000231 and RAP1B and a downregulation of miR-140 level. Silencing of circ_0000231 led to suppression of migration, proliferation, invasion, EMT, and PTX resistance, and induction of apoptosis in PTX-resistant ovarian cancer cells (Liu et al., 2023a). Upregulation of circ_0000231 displayed an opposite effect in PTX-resistant ovarian cancer cells. *In vivo* data also showed that circ_0000231 downregulation enhanced ovarian cancer cell sensitivity to PTX treatment. Further, miR-140 can be sponged by circ_0000231, and target RAP1B in ovarian cancer. Hence, circ_0000231 is vitally involved in chemoresistance of ovarian cancer (Liu et al., 2023a).

#### CircTNPO3

It has been reported that circTNPO3 regulates tumorigenesis in various cancer types. CircTNPO3 inhibited tumor metastasis via decoying IGF2BP3 protein and regulating the expression of Snail and Myc in gastric cancer (Yu et al., 2021). CircTNPO3 suppressed tumor metastasis via interaction with IGF2BP3 and destabilization of SERPINH1 mRNA in clear cell renal cell carcinoma (Pan et al., 2022b). CircTNPO3 enhanced tumor progression via upregulation of STRN expression by decoying miR-199b-5p in hepatocellular carcinoma (Liu and Liu, 2023). CircTNPO3 expression was increased in ovarian cancer tissues and associated with PTX resistance. Downregulation of circTNPO3 increased PTX sensitivity by induction of PTX-mediated apoptosis. CircTNPO3 could decoy miR-1299 and upregulate NEK2 (NIMA-related kinase 2) in ovarian cancer. Hence, circTNPO3 promoted PTX resistance in ovarian cancer (Xia et al., 2020).

#### CircNRIP1

CircNRIP1 was reported to work as a miR-149-5p sponge to target the Akt and mTOR pathways, leading to promoting gastric cancer progression (Zhang et al., 2019b). CircNRIP1 affected the miR-186-5p and MYH9 expressions and enhanced tumor progression and tumor glycolysis in gastric cancer (Liu et al., 2020c). CircNRIP1 sponged miR-629-3p and influenced the PTP4A1 and ERK1/2 pathways, resulting in promotion of cell invasion and migration in cervical cancer (Li et al., 2020b). Inhibition of circNRIP1 reduced the paclitaxel resistance via targeting the miR-211-5p and HOXC8 (homeobox C8) in ovarian cancer (Li et al., 2020c).

### CircRNAs inhibit paclitaxel resistance

CircEXOC6B was observed to repress tumor metastasis via promotion of interaction with RBMS1 (RNA binding motif single strand interacting protein 1) and HuR (human antigen R) in prostate cancer (Zhang et al., 2023b). CircEXOC6B repressed invasion and migration of prostate cancer cells *in vitro* and *in vivo*. CircEXOC6B can bind with RBMS1 and HuR and elevate AKAP12 (A-kinase anchoring protein 12) expression levels, leading to inhibition of tumor metastasis in prostate cancer (Zhang et al., 2023b). In colorectal cancer, circEXOC6B can bind to an mTORC1 activator, RRAGB, reduced tumor progression via antagonization of HIF1A/RRAGB/mTORC1 loop. CircEXOC6B promoted the 5-fluorouracil sensitivity of colorectal cancer cells (Li et al., 2022b). CircEXOC6B decreased tumor progression via sponging miR-421 and inhibiting RUS1 expression in ovarian cancer (Wang et al., 2020c). Inhibition of circEXOC6B facilitated cell invasion and proliferation in ovarian cancer A2780 cells. Overexpression of circEXOC6B displayed an opposite function in ovarian cancer SKOV3 cells via induction of apoptosis and reduction of proliferation and invasion. Further, miR-421 was sponged by circEXOC6B, and RUS1 was a target of miR-421 in ovarian cancer cells (Wang et al., 2020c). The low expression of circEXOC6B was correlated with LNM and younger age in ovarian cancer patients. The high level of circEXOC6B was linked to a better overall survival in ovarian cancer patients (Ning et al., 2018). Zheng et al. reported that circEXOC6B expression levels were downregulated in ovarian cancer specimens, which were correlated with malignant clinical features in ovarian cancer patients. CircEXOC6B reduced PTX resistance and motility and growth in ovarian cancer via targeting miR-376c-3p. FOXO3 (forkhead box O3) was a potential target of miR-376c-3p in ovarian cancer cells. Hence, CircEXOC6B accelerated cell sensitivity to PTX via decoying miR-376c-3p and upregulating FOXO3 in ovarian cancer (Zheng et al., 2022). CircSLC39A8 reduced paclitaxel resistance via modulation of BMF expression by interacting with miR-185-5p in ovarian cancer (Liu et al., 2023b).

### CircRNAs promote cisplatin resistance

#### CircLPAR3

CircLPAR3 was highly expressed in esophageal cancer (ESCC) tissues and cells (Shi et al., 2020; Cheng et al., 2022). ESCC patients with high expression of circLPAR3 had advanced clinical stage and lymph node metastasis. Moreover, circLPAR3 sponged miR-198 and upregulated the expression of MET gene, leading to activation of RAS/MAPK and PI3K/AKT signaling pathways. Furthermore, circLPAR3 enhanced invasion, metastasis and migration in ESCC cells and in mice (Shi et al., 2020). Similarly, another group reported that circLPAR3 downregulation inhibited Warburg effect, metastasis and proliferation and increased cell apoptosis in ESCC cells. Downregulation of circLPAR3 impaired xenograft tumor growth in mice and impeded Warburg effect. CircLPAR3 elevated the expression of LDHA via sponging miR-873-5p in ESCC cells (Cheng et al., 2022). Two studies showed that circLPAR3 expression was higher in oral squamous cell carcinoma (OSCC) samples and cells. Knockdown of circLPAR3 caused suppression of proliferation, stemness and metastasis, and induction of cell apoptosis (Fu et al., 2022a; Su et al., 2022). Mechanistically, circLPAR3 absorbed miR-643 to upregulate the expression of HMGB2. Downregulation of circLPAR3 retarded OSCC oncogenesis in mice (Fu et al., 2022a). Su et al. found that depletion of circLPAR3 reduced glycolysis and repressed tumor progression via suppression of miR-144-3p and promotion of LPCAT1 in OSCC. OSCC patients with higher expression of circLPAR3 displayed a shorter survival (Su et al., 2022).

Chen et al. found that circLPAR3 upregulated the expression of JPT1 via sponging miR-513b-5p, which enhanced glycolytic activation and inhibited radiosensitivity in prostate cancer (Chen et al., 2023b). Liu et al. reported that circLPAR3 increased the cisplatin resistance in ovarian cancer. The expression of circLPAR3 was elevated in cisplatin-resistant ovarian cancer tissues and cells. Downregulation of circLPAR3 led to promotion of cisplatin sensitivity in ovarian cancer cells. Moreover, circLPAR3 interacted with miR-634 and sponged its expression. PDK1 was found to be a target of miR-634 in ovarian cancer. Overexpression of PDK1 inverted the function of miR-634 on cisplatin sensitivity of ovarian cancer. Inhibition of miR-634 rescued circLPAR3 downregulation-mediated cisplatin sensitivity in ovarian cancer. Thereafter, circLPAR3 facilitated cisplatin resistance of ovarian cancer cells (Liu et al., 2022e).

#### CircITGB6

Accumulating evidence has suggested that circITGB6 was involved in cisplatin sensitivity in ovarian cancer. Using circRNA deep sequencing, one group found that circITGB6 expression was upregulated in tumor specimens and serums from ovarian cancer patients, who had cisplatin resistance. The expression of circITGB6 was associated with worse prognosis in ovarian cancer patients (Li et al., 2022c). Upregulation of circITGB6 increased an M2 macrophage-mediated cisplatin resistance *in vitro* and *in vivo*. Mechanistically, circITGB6 was found to bind with IGF2BP2 and FGF9 mRNA to build a ternary complex, including circITGB6, IGF2BP2, and FGF9, in the cytoplasm, contributing to stabilization of FGF9 mRNA and induction of polarization of TAMs to M2 phenotype. Consistently, an antisense oligonucleotide to inhibit circITGB6 expression abrogated the circITGB6-participated cisplatin resistance in ovarian cancer. To sum up, circITGB6 accelerated cisplatin resistance of ovarian cancer cells (Li et al., 2022c).

#### CircPBX3

The expression of circPBX3 was reported to be increased in ovarian tumor samples and cisplatin-resistant ovarian cancer cells. Increased circPBX3 induced apoptosis and promoted colony formation and elevated cisplatin resistance in ovarian cancer cells. Upregulation of circPBX3 increased tumor xenografts growth in mice. Downregulation of circPBX3 inhibited apoptosis and reduced colony formation and cisplatin resistance in ovarian cancer cells. Notably, circPBX3 can bind with IGF2BP2 and elevate the stability of ATP7A at mRNA levels, thereby contributing to upregulation of ATP7A protein in ovarian cancer. The function of circPBX3 upregulation on cisplatin resistance was abolished by downregulation of ATP7A in ovarian cancer cells. In conclude, circPBX3 accelerated cisplatin resistance in ovarian cancer (Fu et al., 2022b).

#### CircSnx12

It has been reported that circSnx12 regulated ferroptosis via sponging miR-224-5p during heart failure (Zheng et al., 2021). Higher expression of circSnx12 was reported in ovarian cancer cells and tumor tissues with cisplatin resistance. Inhibition of circSnx12 caused cell sensitive to cisplatin treatment in cisplatin-resistant ovarian cancer cells via activation of ferroptosis. This phenotype was impaired by suppression of miR-194-5p in cisplatin-resistant ovarian cancer cells. Mechanistical experiments showed that circSnx12 sponged miR-194-5p and subsequently upregulated SLC7A11 in ovarian cancer cells. In conclusion, circSnx12 repressed ferroptosis via targeting miR-194-5p/SLC7A11 axis and conferred cisplatin resistance (Qin et al., 2023b).

#### Circ-PIP5K1A

It has been reported that exosomal circ_PIP5K1A (phosphatidylinositol-4-phosphate 5-kinase type 1 alpha) was highly expressed in NSCLC tumor samples, serum samples and tumor cells (Shao et al., 2021). Moreover, suppression of exosomal circ_PIP5K1A reduced proliferation, invasiveness and migrative abilities in NSCLC cells. Inhibition of circ_PIP5K1A induced apoptosis and cisplatin sensitivity and inhibited tumor growth in mice. Furthermore, circ_PIP5K1A sponged the miR-101 and upregulated the expression of ABCC1 in NSCLC cells (Shao et al., 2021). Suppression of miR-101 reversed the functions of circ_PIP5K1A knockdown on reduction of tumor progression and induction of cisplatin sensitivity. In addition, circ_PIP5K1A promoted NSCLC progression and cisplatin resistance via sponging miR-101 and targeting ABCC1 (Shao et al., 2021). Similarly, another group reported that circ_PIP5K1A increased cisplatin resistance and tumor progression via regulation of miR-493-5p/ROCK1 axis in NSCLC (Feng et al., 2021). DDP-resistant cells and tumor tissues displayed the higher expression of circ_PIP5K1A in NSCLC. Depletion of circ_PIP5K1A decreased cisplatin resistance and cell motility as well as proliferation in DDP-resistant NSCLC cells (Feng et al., 2021). Silencing of circ_PIP5K1A overcame cisplatin resistance via sponging miR-942-5p and targeting NFIB expression in ovarian cancer. The expression of circ_PIP5K1A was elevated in DDP-resistant ovarian cancer cells and tissues. Silencing of circ_PIP5K1A constrained cell invasion, migration and cell growth and increased cisplatin sensitivity in DDP-resistant ovarian cancer cells (Sheng and Wang, 2023). Hence, circ-PIP5K1A promotes cell resistance to cisplatin in ovarian cancer.

#### Circ_0026123

Several studies have revealed the role of circ_0026123 in ovarian cancer progression and drug resistance. For example, one study showed that circ_0026123 expression was upregulated in ovarian cancer cell lines and tissues. Silencing of circ_0026123 reduced migration and proliferation of ovarian cancer cells. Moreover, downregulation of circ_0026123 reduced the expression of CSC markers. Mechanistically, circ_0026123 sponged miR-124-3p and upregulated the expression of EZH2 (enhancer of zeste homolog 2) (Yang et al., 2021). The other study showed that circ_0026123 increased cisplatin resistance and tumor progression of ovarian cancer. The expression of circ_0026123 was increased in DDP-resistant ovarian cancer tissues and cancer cells. Downregulation of circ_0026123 increased cisplatin sensitivity in DDP-resistant ovarian cancer cells. Moreover, downregulation of circ_0026123 suppressed cell migration, angiogenesis, invasion and proliferation in DDP-resistant ovarian cancer cells. Further, circ_0026123 sequestered miR-543 and upregulated RAB1A in ovarian cancer cells. Overexpression of RAB1A or knockdown of miR-543 reversed the functions of circ_0026123 knockdown on cisplatin sensitivity and tumor behaviors in DDP-resistant ovarian cancer cells (Wei et al., 2022). Therefore, circ_0026123 elevated the expression of RAB1A via sponging miR-543, leading to promotion of oncogenesis and cisplatin resistance in ovarian cancer.

#### Circ_0063804

It has been reported that circ_0063804 was involved in cisplatin resistance in ovarian cancer cells. In 108 ovarian cancer patients, circ_0063804 expression was elevated, which was associated with poor prognosis (You et al., 2022). Moreover, circ_0063804 promoted cell proliferation, inhibited cell apoptosis and increased cisplatin resistance in ovarian cancer cells. Notably, circ_0063804 can sponge miR-1276 and the expression of miR-1276 was reduced in ovarian cancer patients. CLU was found to be a target of miR-1276 and was increased in ovarian cancer patients (You et al., 2022). Strikingly, circ_0063804 exacerbated cisplatin resistance and enhanced tumor malignant behaviors via promotion of CLU expression by sponging miR-1276. Furthermore, depletion of circ_0063804 retarded tumor growth, reduced cisplatin resistance in mice (You et al., 2022). In conclusion, circ_0063804 enhances cisplatin resistance in ovarian cancer via targeting miR-1276/CLU axis.

#### Circ_0007841

Several reports showed that circ_0007841 plays a key role in tumorigenesis, including multiple myeloma, NSCLC and ovarian cancer (Wang et al., 2020d; Huang et al., 2021; Long et al., 2022; Guo et al., 2023). Long et al. reported that circ_0007841 took part in tumor progression via regulation of miR-199a-5p/SphK2 axis (Long et al., 2022). Gao et al. found circ_0007841 as a new potential biomarker and drug resistance in multiple myeloma (Gao et al., 2019b). Song et al. reported that circ_0007841 promoted chemotherapy resistance via upregulation of ABCG2 in multiple myeloma (Song et al., 2020). Moreover, one group reported that circ_0007841 promoted tumor malignant progression via targeting miR-338-3p and BRD4 in multiple myeloma (Wang et al., 2020e). Another group found that downregulation of circ_0007841 reduced the development of multiple myeloma and BTZ resistance via inhibition of miR-129-5p and upregulation of JAG1 (Wang et al., 2020d). Huang et al. reported that circ_0007841 accelerated tumorigenesis via facilitating the expression of MEX3C by inhibition of miR-151-3p activity in ovarian cancer cells (Huang et al., 2021). Upregulation of circ_0007841 was observed in ovarian cancer patients, which was associated with poor prognosis. Downregulation of circ_0007841 repressed invasion, migration and cell growth in ovarian cancer. Moreover, circ_0007841 elevated the expression of MEX3C by sponging miR-151-3p (Huang et al., 2021). Downregulation of circ_0007841 led to promotion of cisplatin sensitivity via inhibition of NFIB expression by sponging miR-532-5p in ovarian cancer (Gao and Huang, 2023).

#### Circ_0067934

Numerous studies have shown that circ_0067934 acts as a cancer driver in various types of human cancers (Zhou et al., 2020b; Al-Hawary et al., 2023; Yu et al., 2023). For example, circ_0067934 expression was increased in ESCC (esophageal squamous cell carcinoma) and enhanced cell proliferation (Xia et al., 2016). Circ_0067934 regulated the miR-1324/FZD5/Wnt/β-catenin pathway in hepatocellular carcinoma, which facilitated tumor metastasis and tumor growth (Zhu et al., 2018). Inhibition of circ_0067934 reduced cell invasion, proliferation and migration via regulation of miR-1182 and KLF8 and upregulation of Wnt/β-catenin in NSCLC cells. Circ_0067934 expression was associated with unfavorable prognosis in patients with NSCLC (Wang and Li, 2018; Zou et al., 2018; Zhao et al., 2020). Upregulation of circ_0067934 enhanced cervical cancer progression through targeting miR-545 and EIF3C (Hu et al., 2019). Similarly, overexpression of circ_0067934 facilitated tumor progression via regulation of CXCR1 expression by sponging miR-1304 and associated with poor prognosis in thyroid carcinoma (Wang et al., 2019b; Zhang et al., 2019c). Circ_0067934 reduced tumor cell ferroptosis via modulating miR-545-3p/SLC7A11 pathway and promoted tumor progression via miR-1301-3p/HMGB1 in thyroid cancer (Wang et al., 2021c; Dong et al., 2022). In glioblastoma, overexpression of circ_0067934 conferred tumor progression via activation of PI3K/AKT pathway (Xin et al., 2019). In gastric cancer, downregulation of circ_0067934 contributed to tumor progression via interacting with miR-4705 and regulating BMPR1B expression as well as targeting miR-1301-3p/KIF23 axis (He et al., 2019; Xu et al., 2022). In bladder cancer, circ_0067934 elevated cell invasion, proliferation and migration via inhibition of miR-1304 expression and upregulation of Myc expression (Liu et al., 2020d). In laryngeal squamous, circ_0067934 accelerated tumor progression via interacting with miR-1324 and associated with poor prognosis (Chu, 2020). In breast cancer, circ_0067934 increased the expression of Mcl-1 and performed an oncogenic role in breast tumorigenesis (Wang et al., 2019c). Circ_0067934 was linked to lymph node metastasis and advanced tumor stage. Circ_0067934 facilitated cisplatin resistance via reduction of JNK phosphorylation by targeting miR-545-3p/PPA1 axis in ovarian cancer (Yin et al., 2022).

#### Other circRNAs

Circ_0010467 increased cisplatin resistance via regulation of miR-637, LIF (leukemia inhibitory factor) and STAT3 pathways in ovarian cancer cells, which was induced by AUF1 (Wu et al., 2023b). Circ_0010467 was highly expressed in cisplatin-resistant ovarian cancer cells and tumor tissues as well as serum exosomes, which was linked to poor prognosis of patients with ovarian cancer. Inhibition of circ_0010467 increased cisplatin sensitivity by inhibition of cell proliferation in ovarian cancer (Wu et al., 2023b). Several studies have showed that circFoxp1 promoted tumor progression in gallbladder cancer (Wang et al., 2019d), hepatocellular carcinoma (Wang et al., 2020f), lung cancer (Li et al., 2020d), colon cancer (Luo et al., 2020), renal cell carcinoma (Fang et al., 2021), and osteosarcoma (Zhang et al., 2021c). CircFoxp1 overexpression contributed to cisplatin resistance and cell proliferation in epithelial ovarian cancer. Knockdown of circFoxp1 led to cisplatin sensitivity in ovarian cancer cells and in mice (Luo and Gui, 2020). Exosomal circFoxp1 expression was upregulated in DDP-resistant ovarian cancer patients, which was correlated with IFGO (International Federation of Gynecology and Obstetrics) stage, tumor metastasis and tumor size as well as survival. CircFoxp1 regulated cisplatin resistance in part due to regulation of miR-150-3p and miR-22 in ovarian cancer cells. Moreover, circFoxp1 could regulate the expression of FMNL3 (formin like 3) and CEBPG (CCAAT enhancer binding protein gamma) in ovarian cancer (Luo and Gui, 2020). Hsa_circ_0000585 was reported to increase cisplatin resistance via regulating autophagy in epithelial ovarian cancer (Du et al., 2023).

### CircRNAs inhibit cisplatin resistance

Circ_0078607 has been reported to control tumorigenesis and regulate proliferation and migration in human cancers. Downregulation of circ_0078607 blocked the gastric cancer development and induced the inactivation of the ERK1/2 and Akt pathways via targeting the miR-188-3p and RAP1B (Bian et al., 2021). Circ_0078607 has been found to repress tumor progression via modulating the miR-518a-5p and Fas signaling pathway in ovarian cancer (Zhang et al., 2020c). Moreover, circ_0078607 expression was lower in high-grade serous ovarian cancer and associated with poor prognosis (Zhang et al., 2021d). Furthermore, circ_0078607 was reported to inhibit tumor progression via governing the miR-32-5p and SIK1 pathways in ovarian cancer (Jin and Wang, 2022). Circ_0078607 enhanced cisplatin sensitivity in ovarian cancer cells by regulation of miR-196b-5p and GAS7 (growth arrest-specific 7) expressions (Dai et al., 2023). Circ_0078607 expression was lower in DDP-resistant ovarian cancer cells. Upregulation of circ_0078607 suppressed cisplatin resistance, enhanced apoptosis and repressed cell proliferation in DDP-resistant cells. In term of mechanism, circ_0078607 sequestered miR-196b-5p and elevated the expression of GAS7 (Dai et al., 2023). Therefore, circ_0078607 attenuated cisplatin resistance in ovarian cancer. CircRNA Cdr1as has been reported to inhibit ovarian cancer progression via sponging miR-135b-5p (Chen et al., 2019). CircRNA Cdr1as suppressed cisplatin resistance via sponging miR-1270 and upregulating SCA1 in ovarian cancer (Zhao et al., 2019). Moreover, circRNA Cdr1as inhibited miR-1299 expression and reduced cisplatin-based chemoresistance in ovarian cancer (Wu et al., 2021b).

### CircRNAs regulate docetaxel resistance

Hsa_circ_0006404 and hsa_circ_0000735 have been found to regulate docetaxel (DTX) resistance in ovarian cancer. Hsa_circ_0006404 was remarkably inhibited in DTX-treated ovarian cancer SKOC3-R cells. Circ_0006404 was observed to sponge the miR-346 in ovarian cancer cells. Moreover, miR-346 was observed to inhibit the expression of DKK3 and regulate the p-GP expression in ovarian cancer cells (Chen and Tai, 2022). In addition, Circ_0000735 was found to be increased in DTX-treated ovarian cancer cells. Circ_0000735 can inhibit the expression of miR-526b and elevate the expression of DKK4, leading to regulation of the p-GP expression in ovarian cancer (Chen and Tai, 2022). Hence, overexpression of circRNA_0006404 and inhibition of circRNA_0000735 might be a potential strategy to suppress the expression of p-GP, contributing to better benefit of docetaxel treatment in ovarian cancer.

### CircRNAs regulate 5-fluorouracil resistance

One study showed that circNRIP1 (circRNA nuclear receptor-interacting protein 1) maintained hypoxia-mediated 5-fluorouracil resistance via sponging miR-138-5p and regulating HIF-1alpha-dependent glucose metabolism in gastric cancer (Xu et al., 2020c). Another study revealed that circNRIP1 governed the miR-515-5p/IL-25 axis to regulate cisplatin and 5-fluorouracil resistance in nasopharyngeal carcinoma (Lin et al., 2021). Downregulation of circNRIP1 sensitized cells to 5-fluorouracil treatment via affecting miR-532-3p in colorectal cancer (Liu et al., 2021b). It is unclear whether circNRIP1 regulates the 5-fluorouracil resistance in ovarian cancer.

## Conclusion and future perspectives

**FIGURE 1:**
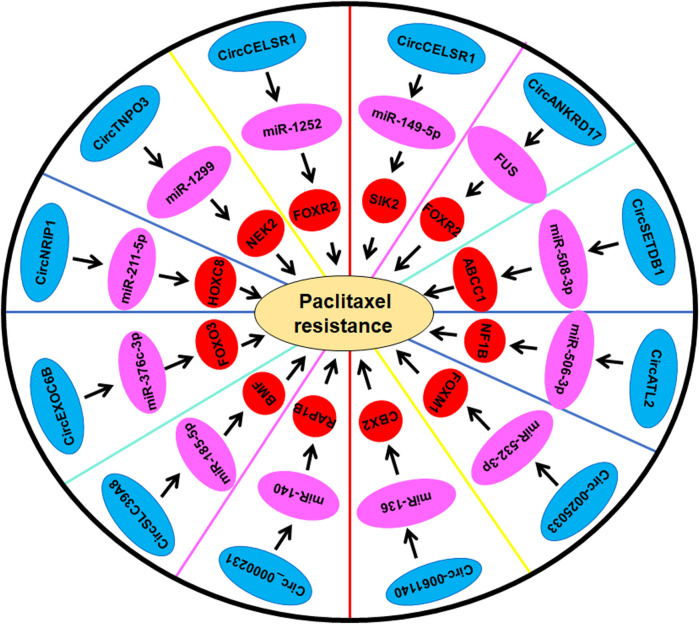
Multiple circRNAs regulate paclitaxel resistance in ovarian cancer.

**FIGURE 2:**
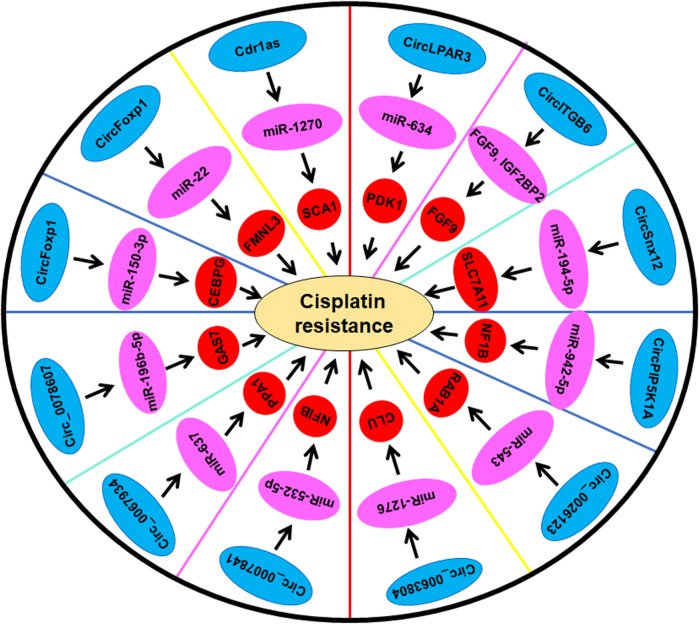
Numerous circRNAs regulate cisplatin resistance in ovarian cancer.

In conclusion, circRNAs play an important role in regulation of paclitaxel resistance (Table 1; Figure 1), cisplatin resistance (Table 2; Figure 2), docetaxel and 5-fluorouracil resistance (Table 3) mainly via sponging miRNAs in ovarian cancer. It is pivotal to mention that there is an interaction between circRNAs and drug resistance in ovarian cancer. Some circRNAs regulate drug resistance in ovarian cancer, while drug resistance changes the expression of certain circRNAs (Qin et al., 2023a). Moreover, circRNAs could be potential biomarkers for prediction of drug resistance in ovarian cancer. Because of critical role of circRNAs in governing drug sensitivity, targeting these circRNAs could be useful for overcoming drug resistance in ovarian cancer.

**TABLE 1:** The role of circRNAs in regulation of paclitaxel resistance.

Item	Mechanism	Target	Function	Ref
CircCELSR1	Sponges miR-1252	FOXR2	Promotes paclitaxel resistance	Zhang et al. (2020a)
CircCELSR1	Sequesters miR-149-5p	SIK2	Increases paclitaxel resistance	Wei et al. (2021a)
CircANKRD17	Interacts with FUS	FOXR2	Contributes to paclitaxel resistance	Liang et al. (2023)
Circ_0061140	Decoys miR-136	CBX2	Enhances paclitaxel resistance	Zhu et al. (2021)
Circ_0025033	Affects miR-532-3p	FOXM1	Induces paclitaxel resistance	Huang et al. (2022)
CircATL2	Sponges miR-506-3p	NFIB	Leads to paclitaxel resistance	Ying et al. (2022)
Circ_0000714	Decoys miR-370-3p	RAB17	Stimulates paclitaxel resistance	Guo et al. (2020)
CircSETDB1	Inhibits miR-508-3p	ABCC1	Enhances paclitaxel resistance	Huang et al. (2023)
Circ_0000231	Represses miR-140	RAP1B	Increases paclitaxel resistance	Liu et al. (2023a)
CircTNPO3	Decoys miR-1299	NEK2	Enhances paclitaxel resistance	Xia et al. (2020)
CircNRIP1	Sponges miR-211-5p	HOXC8	Induces paclitaxel resistance	Li et al. (2020c)
CircEXOC6B	Interacts with miR-376c-3p	FOXO3	Reduces paclitaxel resistance	Zheng et al. (2022)
CircSLC39A8	Decoys miR-185-5p	BMF	Inhibits paclitaxel resistance	Liu et al. (2023b)

**TABLE 2:** The role of circRNAs in regulation of cisplatin resistance.

Item	Mechanism	Target	Function	Ref
CircLPAR3	Sponges miR-634	PDK1	Promotes cisplatin resistance	Liu et al. (2022e)
circITGB6	Binds with IGF2BP2, FGF9	FGF9	Accelerates cisplatin resistance	Li et al. (2022c)
CircPBX3	Binds with IGF2BP2	ATP7A	Enhances cisplatin resistance	Fu et al. (2022b)
circSnx12	Decoys miR-194-5p	SLC7A11	Increases cisplatin resistance	Qin et al. (2023b)
circ_PIP5K1A	Sequesters miR-942-5p	NFIB	Induces cisplatin resistance	Sheng and Wang (2023)
circ_0026123	Interacts with miR-543	RAB1A	upregulates cisplatin resistance	Wei et al. (2022)
circ_0063804	Binds with miR-1276	CLU	Elevates cisplatin resistance	You et al. (2022)
circ_0007841	Sponges miR-532-5p	NFIB	Promotes cisplatin resistance	Gao and Huang (2023)
circ_0067934	Decoys miR-543-3p	PPA1, JNK	Facilitates cisplatin resistance	Yin et al. (2022)
Circ_0010467	Sequesters miR-637	LIF, STAT3	Induces cisplatin resistance	Wu et al. (2023b)
CircFoxp1	Binds with miR-22, miR-150-3p	FMNL3, CEBPG	Enhances cisplatin resistance	Luo and Gui (2020)
Circ_0078607	Sponges miR-196b-5p	GAS7	Inhibits cisplatin resistance	Dai et al. (2023)
Cdr1as	Decoys miR-1270	SCA1	Suppresses cisplatin resistance	Zhao et al. (2019)

**TABLE 3:** The role of circRNAs in regulation of resistance to docetaxel and 5-fluorouracil.

Item	Mechanism	Target	Function	Ref
Circ_0006404	Sponges miR-346	DKK3	Regulates docetaxel resistance in ovarian cancer	Chen and Tai (2022)
Circ_0000735	Inhibits miR-526b	DKK4	Regulates docetaxel resistance in ovarian cancer	Chen and Tai (2022)
CircNRIP1	Sponging miR-138-5p	HIF-1alpha	Induces 5-fluorouracil resistance in gastric cancer	Xu et al. (2020c)
CircNRIP1	Sponging miR-515-5p	IL-25	Controls 5-fluorouracil resistance in nasopharyngeal carcinoma	Lin et al. (2021)
CircNRIP1	Sponging miR-532-3p	N/A	Induces 5-fluorouracil resistance in colorectal cancer	Liu et al. (2021b)

**FIGURE 3:**
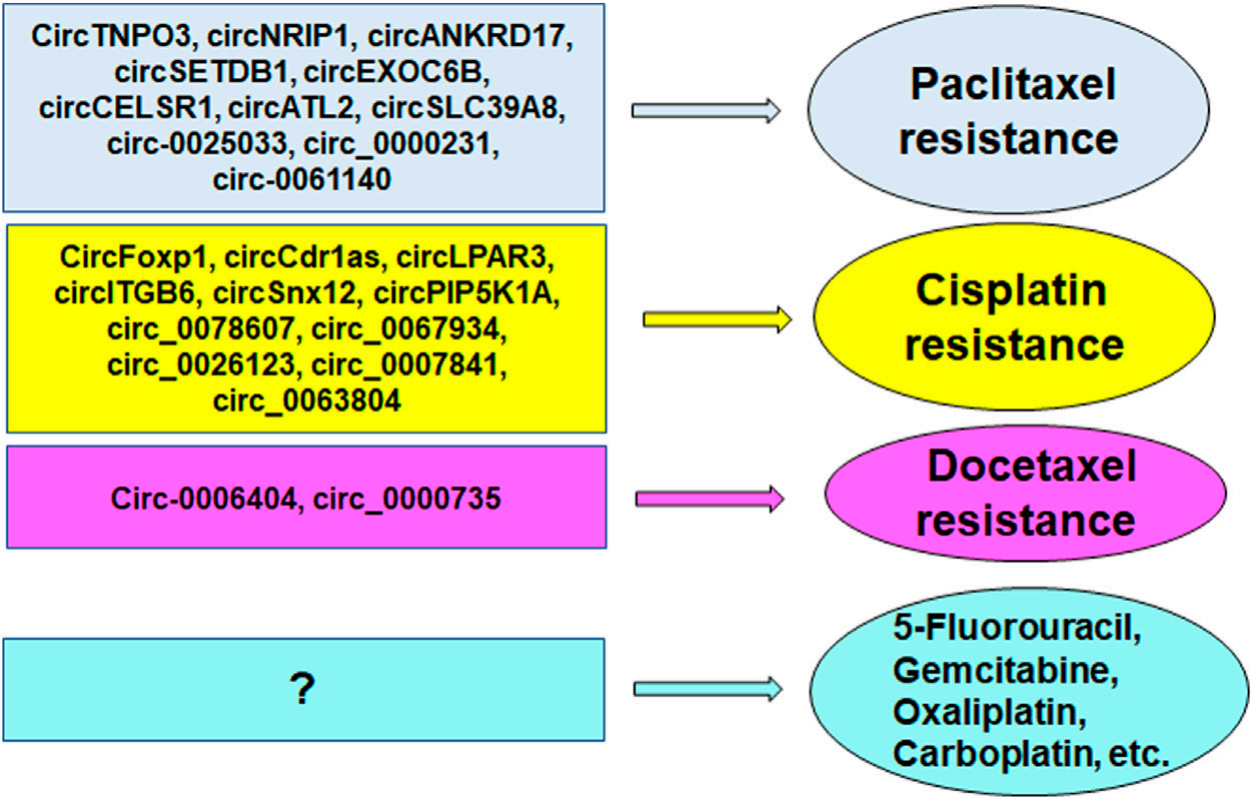
CircRNAs regulate drug resistance in ovarian cancer, including cisplatin, paclitaxel and docetaxel.

To fully understand the mechanism of drug resistance in ovarian cancer, several issues should be mentioned. A majority of circRNAs promote drug resistance in ovarian cancer, including cisplatin, paclitaxel and docetaxel (Figure 3). A few circRNAs increase drug sensitivity in ovarian cancer. Besides circRNAs, miRNAs and lncRNAs are critically involved in modulation of drug resistance in ovarian cancer. Three chemotherapeutic drugs, cisplatin, docetaxel and paclitaxel, were reported to be regulated by circRNAs in ovarian cancer. It is interesting to explore whether resistance to other chemotherapeutic agents, such as gemcitabine, oxaliplatin, carboplatin, is regulated by circRNAs in ovarian cancer. It is important to note that besides noncoding RNAs, protein post-translational modifications regulate drug resistance in human cancers, including ovarian cancer. The E3 ubiquitin ligases regulated tumor progression and immunotherapy resistance in human cancer (Hou et al., 2023). Hence, targeting the E3 ubiquitin ligases might be helpful for overcoming drug resistance in ovarian cancer.

Several compounds have shown to regulate the expression of circRNAs in human cancers. Curcumin, an active component of turmeric, increased the expression of circ-PLEKHM3 in ovarian cancer, and targeted miR-320a/SMG1 axis (Sun and Fang, 2021). Moreover, curcumin has been reported to be a therapeutic agent in cancer therapy via affecting circRNAs (Si et al., 2023). It is necessary to determine whether curcumin could enhance drug sensitivity via targeting circRNAs in ovarian cancer. In addition, it is unclear whether there is the feedback regulation between circRNAs and drug resistance in ovarian cancer. It is elusive whether circRNAs interact with other factors involved in drug sensitivity to modify chemoresistance. Cancer stem cells play an essential role in inducing drug resistance. It is required to determine whether circRNAs control drug resistance via targeting ovarian cancer stem cells. Importantly, the application of circRNAs for prediction and treatment in clinic remain inadequate and challenging. In summary, in-depth exploration into the mechanisms of drug resistance in ovarian cancer is essential to development novel drugs and treatment strategies by targeting ncRNAs that can overcome drug resistance.

## Figures and Tables

**Table:** 

**ABCC1**	ATP-binding cassette subfamily C member 1
**AKAP12**	A-kinase anchoring protein 12
**ANKRD17**	ankyrin repeat domain 17
**ATG13**	autophagy related 13
**ATL2**	Atlastin GTPase 2
**ATP2B4**	ATPase plasma membrane Ca2+ transporting 4
**ATRNL1**	attractin-like 1
**BMF**	Bcl-2 modifying factor
**BNC2**	basonuclin zinc finger protein 2
**BRCA1**	breast cancer 1
**BRD4**	bromodomain containing 4
**BTZ**	Barentsz
**CBX2**	chromobox 2
**CCK-8**	cell counting kit-8
**CDH1**	E-cadherin
**CDK6**	cyclin dependent kinase 6
**CEBPG**	CCAAT enhancer binding protein gamma
**CELSR1**	cadherin EGF LAG seven-pass G-type receptor 1
**CERS6**	ceramide synthase 6
**CHD2**	chromodomain helicase DNA binding protein 2
**CircEXOC6B**	circRNA exocyst complex component 6B
**circRNAs**	circular RNAs
**circMFN2**	circRNA mitofusin 2
**circNRIP1**	circRNA nuclear receptor-interacting protein 1
**circSETDB1**	circRNA SET domain bifurcated histone lysine methyltransferase 1
**COL1A1**	collagen type 1 alpha 1 chain
**CUL4B**	cullin 4B
**DKK3**	dickkopf WNT signaling pathway inhibitor 3
**DNAJB6**	DnaJ homolog subfamily B member 6
**DSC1**	desmocollin-1
**EMT**	epithelial-mesenchymal transition
**ERK1/2**	extracellular signal-regulated kinase 1/2
**EXOC6B**	exocyst complex component 6B
**EZH2**	enhancer of zeste homolog 2
**FAM13B**	family with sequence similarity 13 member B
**FBXW7**	F-box and WD repeat domain containing 7
**FGF9**	fibroblast growth factor 9
**FGFR3**	fibroblast growth factor receptor 3
**FMNL3**	formin like 3
**FOXC1**	forkhead box C1
**FOXM1**	forkhead box M1
**FOXN2**	forkhead box N2
**FOXR2**	forkhead box R2
**FZD5**	frizzled class receptor 5; HMGB1: high mobility group box 1
**GAS7**	growth arrest-specific 7
**hTERT**	human telomerase reverse transcriptase
**HOXC8**	homeobox C8
**HuR**	human antigen R
**IFNGR2**	interferon gamma receptor 2
**IGF2BP2**	insulin like growth factor 2 mRNA binding protein 2
**ITCH**	Itchy E3 ubiquitin protein ligase
**ITGB6**	integrin subunit beta 6
**JAG1**	Jagged-1
**JPT1**	Jupiter microtubule associated homolog 1
**JNK**	c-jun N-terminal kinase
**PPA1**	inorganic pyrophosphatase 1
**KLF4**	kruppel-like factor 4
**KRT7**	keratin 7
**lncRNAs**	long noncoding RNAs
**LDHA**	lactate dehydrogenase A
**LETM1**	leucine zipper and EF-hand containing transmembrane protein 1
**MAP3K3**	mitogen-activated protein kinase kinase kinase 3
**MAPK**	mitogen-activated protein kinase 1
**MDM2**	mouse double minute 2
**MEX3C**	mex-3 RNA binding family member C
**miRNAs**	microRNAs; ncRNAs
**mTORC1**	mammalian target of rapamycin C1
**MUC**	mucin; noncoding RNAs
**MYO6**	myosin-VI; TNPO3: transportin-3
**NEAT1**	nuclear paraspeckle assembly transcript 1
**NEK2**	never in mitosis gene a-related kinase 2
**NFIB**	nuclear factor I B
**NRIP1**	nuclear receptor interacting protein 1
**N4BP2L2**	NEDD4 binding protein 2 like 2
**OSCC**	oral squamous cell carcinoma
**PBX3**	pre B cell leukemia homeobox 3
**PD1**	programmed cell death 1
**PDK1**	pyruvate dehydrogenase kinase 1
**PHC3**	polyhomeotic homolog 3
**PI3K**	phosphoinositide 3-kinase
**PIP5K1A**	phosphatidylinositol-4-phosphate 5-kinase type 1 alpha
**piRNAs**	piwi-interacting RNAs
**PLEKHM3**	pleckstrin homology domain containing M3
**PRPF8**	pre-mRNA processing factor 8
**PTEN**	phosphatase and tensin homolog
**PTP4A1**	protein tyrosine phosphatase 4A1
**PSAT1**	phosphoserine aminotransferase 1
**PUM1**	pumilio RNA binding family member 1
**PVT1**	plasmacytoma variant translocation
**RAB11FIP1**	RAB11 family interacting protein 1
**RAB17**	Ras-related protein 17; RB: retinoblastoma
**RASA1**	RAS p21 protein activator 1
**RASSF8**	RAS association domain family member 8
**RBMS1**	RNA binding motif single strand interacting protein 1
**RHOBTB3**	Rho related BTB domain containing 3
**ROCK1**	Rho-associated coiled-coil containing protein kinase 1
**RRAGB**	Ras related GTP binding B
**rRNAs**	ribosomal RNAs; SH2D3A
**SERPINH1**	serpin family H member 1
**SFRP1**	secreted frizzled related protein 1
**SH2**	domain containing 3A
**SIK2**	salt inducible kinase 2
**siRNAs**	small interfering RNAs
**SLC3A2**	solute carrier family 3 member 2
**SLC1A5**	solute carrier family 1 member 5
**Snx12**	sorting nexin 12
**snoRNAs**	small nucleolar RNAs
**SOX9**	SRY-box transcription factor 9
**SphK2**	sphingosine kinase 2
**SREBF1**	sterol regulatory element binding transcription factor 1
**STAT3**	signal transducer and activator of transcription 3
**STRN**	striatin
**TAMs**	tumor-associated macrophages
**UBAP2**	ubiquitin associated protein 2
**WHSC1**	Wolf-Hirschhorn syndrome candidate 1
**ZEB1**	Zinc finger E-box-binding homeobox 1
**ZNF608**	zinc finger protein 608
